# Pulmonary cryptococcosis with or without immunocompromise: a retrospective comparative study of clinical and CT features and influential factors for lesion absorption

**DOI:** 10.3389/fmed.2026.1935368

**Published:** 2026-08-28

**Authors:** Juan Liao, Wenzhao Zhang, Liqing Peng, Ying Liu, Dianying Liao, Jianqun Yu

**Affiliations:** 1 Department of Medical Imaging, People’s Hospital of Shifang City, Shifang, China; 2 Department of Radiology, West China Hospital, Sichuan University, Chengdu, China; 3 Pathology Department, West China Hospital, Sichuan University, Chengdu, China

**Keywords:** antifungal therapy, chest imaging, immune status, pulmonary cryptococcosis, serum cryptococcal antigen

## Abstract

**Background:**

Pulmonary cryptococcosis (PC) is an invasive fungal infection with rising incidence in immunocompetent populations. The differential clinical and imaging characteristics between immunocompromised (ICP) and non-immunocompromised (NICP) patients remain incompletely characterized, limiting precision in diagnosis and management.

**Method:**

A retrospective study was conducted on 117 patients with confirmed PC from a single tertiary center. Patients were stratified into ICP and NICP groups based on refined criteria. Clinical symptoms, laboratory findings, and chest CT imaging features were systematically compared. Quantitative volumetric analysis was performed on follow-up CT scans (*n* = 31) to calculate the degree of absorption (DOA). LASSO regression was used for exploratory prognostic factor analysis.

**Result:**

A high proportion of patients (59.0%) were asymptomatic at presentation. After False Discovery Rate (FDR) correction, no significant differences in clinical symptoms were found between ICP and NICP groups. Laboratory profiles, however, provided objective differentiation, with ICP patients showing significantly lower red blood cell counts and albumin levels (all Q < 0.05). CT imaging revealed that solitary nodules (Type I) were more common in NICP patients (Q = 0.030), while complex patterns like patchy consolidation (Type III) and numerous lesions (≥10) were significantly associated with ICP status (Q = 0.035). The halo sign was not a robust differentiator after FDR correction (Q = 0.105). Serum CrAg testing showed high diagnostic accuracy (83.9% positivity). In follow-up, a significantly lower DOA was observed in ICP patients (75.1% vs. 86.7%, *P* = 0.038). The conservative LASSO model identified no robust predictors of DOA, but an exploratory analysis nominated imaging type, immune status, and treatment duration as candidate factors.

**Conclusions:**

The clinical presentation of PC is non-specific and cannot reliably differentiate immune status, underscoring the necessity of integrated laboratory and radiographic evaluation. ICP patients present with distinct laboratory abnormalities and more complex imaging patterns. Treatment response assessment should combine clinical, radiographic, and serological trends, as antigen persistence is common. From a clinical perspective, population-based screening is not recommended given the high proportion of incidentally discovered cases; instead, diagnostic vigilance during routine imaging should be emphasized. In immunocompetent patients with small solitary lesions, surgery alone represents an effective option that can reduce the need for prolonged antifungal therapy. Larger prospective studies are needed to validate the exploratory prognostic factors identified.

## 1 Introduction

Pulmonary cryptococcosis (PC) is typically contracted by inhaling dry yeast cells or spores through the respiratory tract, resulting in primary pulmonary infection (1). It was previously believed that pulmonary cryptococcosis was often an opportunistic infection in immunocompromised patients (2). Currently, studies have found that the incidence rate of patients with no risk factors and normal immune function is more than 50%, and it is increasing (3). The clinical symptoms of PC lack specificity, and the imaging features are difficult to distinguish from those of primary lung cancer, tuberculoma, or metastatic lung tumors, leading to missed diagnoses and misdiagnoses (4, 5). Currently, the diagnosis of pulmonary cryptococcosis typically relies on clinical and radiological manifestations, as well as laboratory confirmation (3). The detection of serum cryptococcus capsule antigen (CrAg) is helpful for the early diagnosis of cryptococcosis, but it cannot be used to evaluate the treatment response to pulmonary cryptococcal infection (6). Imaging examinations are the primary method for monitoring and diagnosing pulmonary diseases, as well as the main criteria for evaluating the clinical treatment outcomes of PC. It has been reported that the time for complete resolution of imaging after antifungal treatment ranges from 2 to 18 months, with most residual lung abnormalities (7). The factors influencing imaging absorption are currently unclear. Previous research on PC has primarily focused on normal immune function or immunocompromised states. There are few comparative studies on the clinical and imaging features under different immune states, and these studies have typically involved small sample sizes (8). To address this gap,this study retrospectively collected clinical and CT data from 117 patients diagnosed with cryptococcosis through histopathology at our hospital from January 2014 to September 2023. The clinical and imaging characteristics of PC, as well as the relevant factors influencing image absorption under different immune states, were analyzed to enhance understanding of this disease.

## 2 Materials and methods

### 2.1 Subjects

A total of 331 patients diagnosed with pulmonary cryptococcosis at West China Hospital of Sichuan University from 2014 to 2023 were retrospectively screened, and 117 patients were finally enrolled according to predefined inclusion and exclusion criteria.

The inclusion criteria are as follows: ① meeting the standards outlined in the 2010 Chinese Expert Consensus on the Diagnosis and Treatment of Cryptococcal Infections (9), with positive histopathological examination, or positive culture from sterile cavity fluid or fungal specimens; ② obtaining lung tissue pathology through CT-guided percutaneous biopsy, fiberoptic bronchoscopy biopsy, surgical procedures, etc.; ③ having complete medical record information; ④ approval from the Medical Ethics Committee; ⑤ having obtained chest CT images before treatment.

Exclusion criteria include: ① patients with a lack of chest CT examination or unqualified images; ② incomplete clinical case data.

The patients were divided into two groups based on their immune status. Immunocompromised patients (ICP) were defined as those meeting one or more of the following criteria (10): (1) receipt of systemic immunosuppressive therapy within 3 months prior to diagnosis, including corticosteroids (≥10 mg prednisone equivalent per day for ≥2 weeks), chemotherapeutic agents for malignancies, or biological/other immunosuppressants (e.g., TNF-α inhibitors, calcineurin inhibitors, antimetabolites); (2) HIV infection with CD4+ T-cell count <200 cells/*μ*L or primary immunodeficiency disorders; (3) presence of specific severe comorbidities, including hematological malignancies, solid organ transplantation, end-stage renal disease requiring maintenance dialysis, or chronic liver disease of Child-Pugh grade B or C. Based on these criteria, 49 patients (41.9%, 49/117) were classified into the ICP group. The remaining 68 patients (58.1%, 68/117), who did not meet any of the above criteria, were classified as non-immunocompromised (NICP).

### 2.2 Clinical information

Baseline clinical information was systematically collected, including age, sex, clinical symptoms, and serial laboratory indicators at initial diagnosis and post-treatment follow-up, including routine blood tests, C-reactive protein, procalcitonin, serum cryptococcal antigen (CrAg), and CrAg titers.

Histopathological diagnosis was performed on lung biopsy or resected specimens. Routine hematoxylin-eosin (HE) staining and specific fungal staining, including Mucicarmine and Grocott-Gomori's methenamine silver (GMS) staining, were applied to identify fungal morphological features. Detailed histopathological manifestations are presented in the Results section.

Serum fungal biomarkers, including 1,3-*β*-D-glucan (BG), galactomannan (GM), and CrAg, were detected in the Department of Laboratory Medicine, West China Hospital. Due to the decade-long study period (2014–2023), detection platforms and commercial kits were periodically updated:

(1) 1,3-*β*-D-glucan: Turbidimetric kinetic horseshoe crab lysate assay (2014–2019); magnetic chemiluminescence immunoassay (CLIA, Dynamiker Biotechnology, Tianjin, China) after 2021, with parallel validation during 2020–2021.
(2) Galactomannan: Platelia Aspergillus ELISA kit (Bio-Rad, France, cutoff = 0.5) from 2014 to 2021; Dynamiker CLIA kit from 2022 onward.
(3) Cryptococcal capsular antigen: IMMY latex agglutination assay (2014–2018); IMMY CrAg lateral flow assay combined with quantitative Dynamiker CLIA detection after 2019.

All laboratory tests were strictly performed in accordance with the manufacturer's official guidelines.

Antifungal treatment was initiated based on the 2010 Chinese Expert Consensus on Cryptococcal Infections and individualized according to immune status, disease severity, and drug tolerance. Standard regimens included fluconazole monotherapy (400 mg daily), voriconazole monotherapy (200 mg twice daily), and azole combined with flucytosine(1 g/day) for severe or refractory cases. For asymptomatic immunocompetent patients with solitary small lesions, surgical resection alone was adopted without routine antifungal medication. The duration of antifungal therapy was planned to range from 6 to 12 months, with adjustments made according to clinical response, radiographic improvement, and changes in CrAg titers.

### 2.3 Imaging data acquisition and image analysis

Philips (Brilliance 64, Philips Netherlands) and Siemens (Siemens Somatom Definition FLASH, Sensation 16, Siemens Germany) CT scanners were used for image acquisition. The scanning parameters were as follows: tube voltage 120–140 kV, tube current 100–210 mAs, matrix 512 × 512, covering from the apex to the bottom of the lungs, with a layer thickness of 5 mm and a layer spacing of 5 mm, and a 1 mm thin-layer high-resolution reconstruction. In this group of cases, 80 patients underwent a plain scan, while 37 patients underwent both a plain scan and an enhanced scan. The enhanced scan was performed using a non-ionic iodine contrast agent (Ioversol Injection, 350 mg/mL, dosage of 1.5 mL/kg) and a high-pressure syringe for intravenous injection. The injection was administered into the anterior elbow vein at a rate of 3.0 mL/s.

All images were individually analyzed by a senior radiologist, who observed the size, shape, density, internal structure (cavity, air bronchogram, calcification), edges (lobulation sign, spicule sign), boundaries (blurred, clear), and changes in adjacent structures (halo sign, vascular cluster sign, pleural indentation sign) of the lesion. According to the morphology and distribution of lesions on chest CT scans, pulmonary cryptococcosis were divided into four types (11):1) Nodular imaging features with clear boundaries are classified into solitary nodule/mass type (Type I) and multiple nodule/mass type (Type II). 2) Unclear lung infiltration is classified as patchy consolidation type (Type III). 3) The simultaneous appearance of nodular shadows and patchy infiltration is classified as mixed type (Type IV).

Quantitative volumetric analysis was performed for the 31 cases with follow-up CT after antifungal treatment using the Dr. Wise® AI-based medical imaging analysis platform (V1.0.1.1, DeepWise, China). Two blinded cardiothoracic radiologists independently verified the automated segmentation results. The system automatically identified and segmented pulmonary lesions using deep learning algorithms, providing precise volume measurements (in cm³) for each lesion. The degree of absorption (DOA) was calculated using the formula: DOA = (1 - V_post/V_pre) × 100%, where V_pre represents the baseline volume and V_post represents the post-treatment volume. This DOA value represents the percentage reduction in lesion volume after treatment, with higher values indicating better treatment response and greater lesion absorption. Inter-observer reproducibility for volume measurements was excellent, with an intraclass correlation coefficient (ICC) of 0.98 (95% CI: 0.95–0.99) based on reassessment of a 20-case subset.

### 2.4 Statistical analysis

Statistical software SPSS 27.0 and R language (4.3.1) were used for data processing, and the quantitative data were presented as the mean ± standard deviation (mean ± SD). When the distribution was normal and the variance was homogeneous, t-test analysis was used to compare the two groups. If the distribution did not adhere to the normal distribution, the reciprocal transformation was applied to convert it to a normal distribution and recalculate it. The logarithmic transformation of antigen titers was conducted using the Wilcoxon rank sum test. Count data was expressed as [n (%)], and intergroup comparisons were made using the *χ*2 test or Fisher's exact probability method. To control the false discovery rate (FDR) in multiple hypothesis testing, the Benjamini-Hochberg procedure was applied to all univariate comparisons, with an FDR-adjusted Q-value < 0.05 considered statistically significant. Given the small follow-up cohort (*n* = 31), LASSO regression was applied for exploratory feature selection. The conservative ‘lambda.1se’ criterion—which selects the model within one standard error of the minimum cross-validated error—was used with 10-fold cross-validation to mitigate overfitting. The failure of the ‘lambda.1se’ model to select any variables suggests that no strong associations were detectable at this sample size. Any findings from the ‘lambda.min’ model are considered hypothesis-generating. To address the potential confounding effect of heterogeneous treatment regimens on clinical outcomes, a binary variable—"antifungal treatment” (including medical therapy alone and surgery combined with antifungal therapy) versus “surgery alone without antifungal treatment” —was created and forced into the multivariate logistic regression models as a covariate, irrespective of its univariate significance. This approach ensured that the independent effects of imaging type and immune status on lesion absorption were estimated after adjusting for treatment exposure. For all statistical tests, a *p*-value of less than 0.05 indicated a significant difference. The statistical methods were reviewed and validated by a senior researcher with expertise in biostatistics from our institution.

## 3 Results

### 3.1 Clinical characteristics of pulmonary cryptococcus

**Figure 1:**
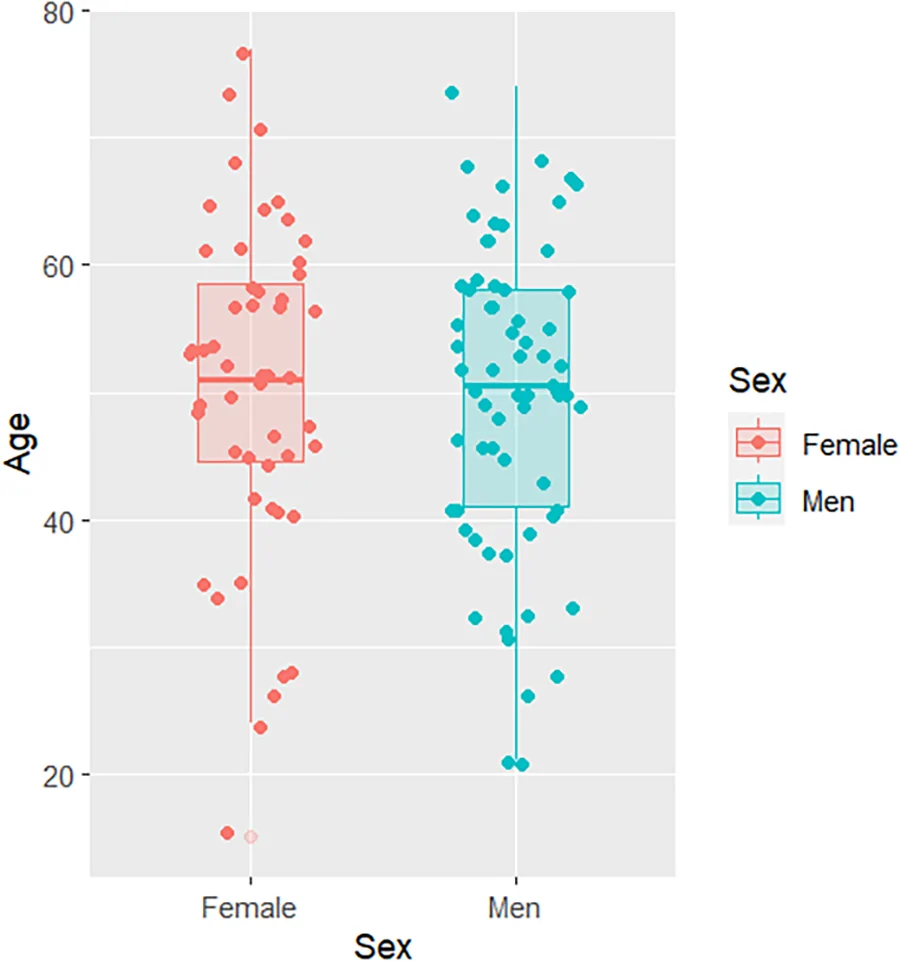
Distribution of age and gender at onset of pulmonary cryptococcosis.

A total of 117 patients with histopathologically confirmed PC were enrolled, including 66 males (56.4%) and 51 females (43.6%). The mean age was 50.06 ± 12.43 years (range: 15–77 years), with a median of 51 years; 55 patients (47.0%) were under 50 years of age (Figure 1). Regarding lifestyle and environmental exposure, 18 patients (15.4%) had a history of smoking, and an equal number had a history of alcohol consumption. Only two patients (1.7%) reported a clear history of exposure to specific environments: one had contact with pigeons and prolonged exposure to humid soil, and the other had contact with poultry (chickens).

All 117 patients were diagnosed by histopathological examination of lung tissue specimens obtained via surgical resection (57.3%, 67/117), CT-guided percutaneous lung biopsy (41.0%, 48/117), or bronchoscopic lung biopsy (1.7%, 2/117). Histopathological examination revealed granulomatous inflammation in 81.2% (95/117) and chronic inflammation in 18.8% (22/117). Special staining results were as follows: hexamine silver staining was positive in all 117 cases; PAS staining was positive in 106 cases; mucicarmine staining was positive in 44 cases, partially or individually positive in 39 cases, and negative in 14 cases; acid-fast staining was negative in 104 cases.

Regarding immune status, 49 patients (41.9%) were classified as immunocompromised (ICP), with causes including diabetes mellitus (17.1%, 20/117), rheumatoid arthritis (6.8%, 8/117), chronic hepatitis B (5.1%, 6/117), liver dysfunction (5.1%, 6/117), kidney transplantation (3.4%, 4/117), and others. The remaining 68 patients (58.1%) had no identifiable immunocompromising conditions (NICP).

Clinically, 48 patients (41.0%) presented with respiratory or systemic symptoms, including cough, sputum production, hemoptysis, chest tightness, tachypnea, asthma, dyspnea, chest pain, fever, and night sweats. Symptomatic patients accounted for 49.0% (24/49) of the ICP group and 35.3% (24/68) of the NICP group. Although univariate analysis suggested differences in the prevalence of cough, asthma, and dyspnea between the two groups (*P* < 0.05), none of these symptoms remained statistically significant after False Discovery Rate (FDR) correction (all Q > 0.05), as detailed in Table 1. These findings indicate that baseline clinical presentation was comparable between the two groups.

**Table 1:** Comparison of general clinical characteristics with PC between ICP and NICP.

Characteristic	ICP (*n* = 49)	NICP (*n* = 68)	Test statistic	*P* value	Q-Value
Age, mean (range), years	52.18 (26–77)	48.53 (15–71)	t = −1.578	0.117	0.234
Gender(male/female)	27/22	39/29	*χ*² = 0.059	0.809	0.809
Contact history, *n*	2	0	χ² = 2.824	0.093	0.223
Cough, *n*	22	17	χ² = 5.558	**0** **.** **018**	0.108
sputum, *n*	15	11	χ² = 3.434	0.064	0.192
Hemoptysis, *n*	1	2	χ² = 0.092	0.761	0.809
Chest tightness, *n*	2	1	χ² = 0.777	0.378	0.567
Polypnea, *n*	1	3	χ² = 0.485	0.486	0.648
Asthma, *n*	5	1	χ² = 4.465	**0**.**035**	0.140
Dyspnea, *n*	4	0	χ² = 5.748	**0**.**017**	0.108
Chest pain, *n*	5	6	χ² = 0.083	0.773	0.809
Fever, *n*	3	1	χ² = 1.931	0.165	0.282

ICP, mmunocompromised patients; NICP, non-immunocompromised patients. Q-values were adjusted for multiple comparisons using the Benjamini-Hochberg false discovery rate (FDR) method. A Q-value < 0.05 was considered statistically significant.

### 3.2 Laboratory findings

The laboratory findings at diagnosis are presented in Table 2. After False Discovery Rate (FDR) correction for multiple comparisons, three indicators remained significantly different between the ICP and NICP groups: the ICP group showed lower red blood cell counts, serum total protein, and serum albumin levels (all Q < 0.05). These findings are consistent with the potential impact of underlying immunocompromising conditions on nutritional status and hematological parameters. No other significant differences were observed between the two groups (*P* > 0.05).

**Table 2:** Comparison of laboratory test values between ICP and NICP groups.

Parameter	ICP(*N* = 49)	NICP(*N* = 68)	*χ* ^2^	*P* value	Q-value
Low red blood cell count, *n* (%)	12 (24.5%)	5 (7.4%)	6.734	**0** **.** **009**	**0**.**028**
Low serum albumin, *n* (%)	12 (24.5%)	4 (8.3%)	8.353	**0**.**004**	**0**.**028**
High white blood cell count, *n* (%)	4 (8.2%)	6 (8.8%)	0.016	0.900	0.945
Low absolute neutrophil count, *n* (%)	2 (4.1%)	0 (0%)	2.824	0.093	0.217
Low absolute lymphocyte count, *n* (%)	14 (28.6%)	11 (16.2%)	2.604	0.107	0.217
Low eosinophil count, *n* (%)	6 (12.2%)	5 (7.4%)	0.800	0.371	0.562
High erythrocyte sedimentation rate, *n* (%)	9 (36.0%, 9/25)	3 (21.4%, 3/14)	0.895	0.344	0.562
High C-reactive protein, *n* (%)	7 (35.0%, 7/20)	5 (33.3%, 5/15)	0.011	0.918	0.945
High procalcitonin, *n* (%)	10 (47.6%, 10/21)	6 (40.0%, 6/15)	0.206	0.650	0.758
High interleukin−6 levels, *n* (%)	5 (31.3%, 5/16)	2 (20.0%, 2/10)	0.386	0.529	0.679
Low serum total protein, *n* (%)	20 (40.8%)	19 (27.9%)	2.124	0.145	0.246
Low albumin levels, *n* (%)	14 (28.6%)	7 (10.3%)	6.460	**0**.**011**	**0**.**039**
Low CD4 count, *n* (%)	11 (44.0%, 11/25)	6 (42.9%, 6/14)	0.005	0.945	0.945
Low CD4/CD8 ratio, *n* (%)	8 (32.0%, 8/25)	4 (28.6%, 4/14)	0.050	0.824	0.945

ICP, immunocompromised patients; NICP, non-immunocompromised patients. Q-values were adjusted for multiple comparisons using the Benjamini–Hochberg false discovery rate (FDR) method. A Q-value < 0.05 was considered statistically significant and is shown in bold.

In addition, 29 patients underwent serum galactomannan (GM) testing, and 33 patients underwent serum (1,3)-β-D-glucan (BG) testing, all of which were negative. Among 21 patients screened for tuberculosis interferon-*γ* release assay, only one case was positive. All enrolled patients had negative serum HIV antibodies. Twenty-three patients underwent lumbar puncture to collect cerebrospinal fluid samples. Among these, two cases showed positive CSF cryptococcal antigen, and two cases showed elevated CSF protein. All sputum fungal smears/cultures (36 cases) and blood cultures (19 cases) were negative.

### 3.3 CT features of pulmonary cryptococcosis

**Figure 2:**
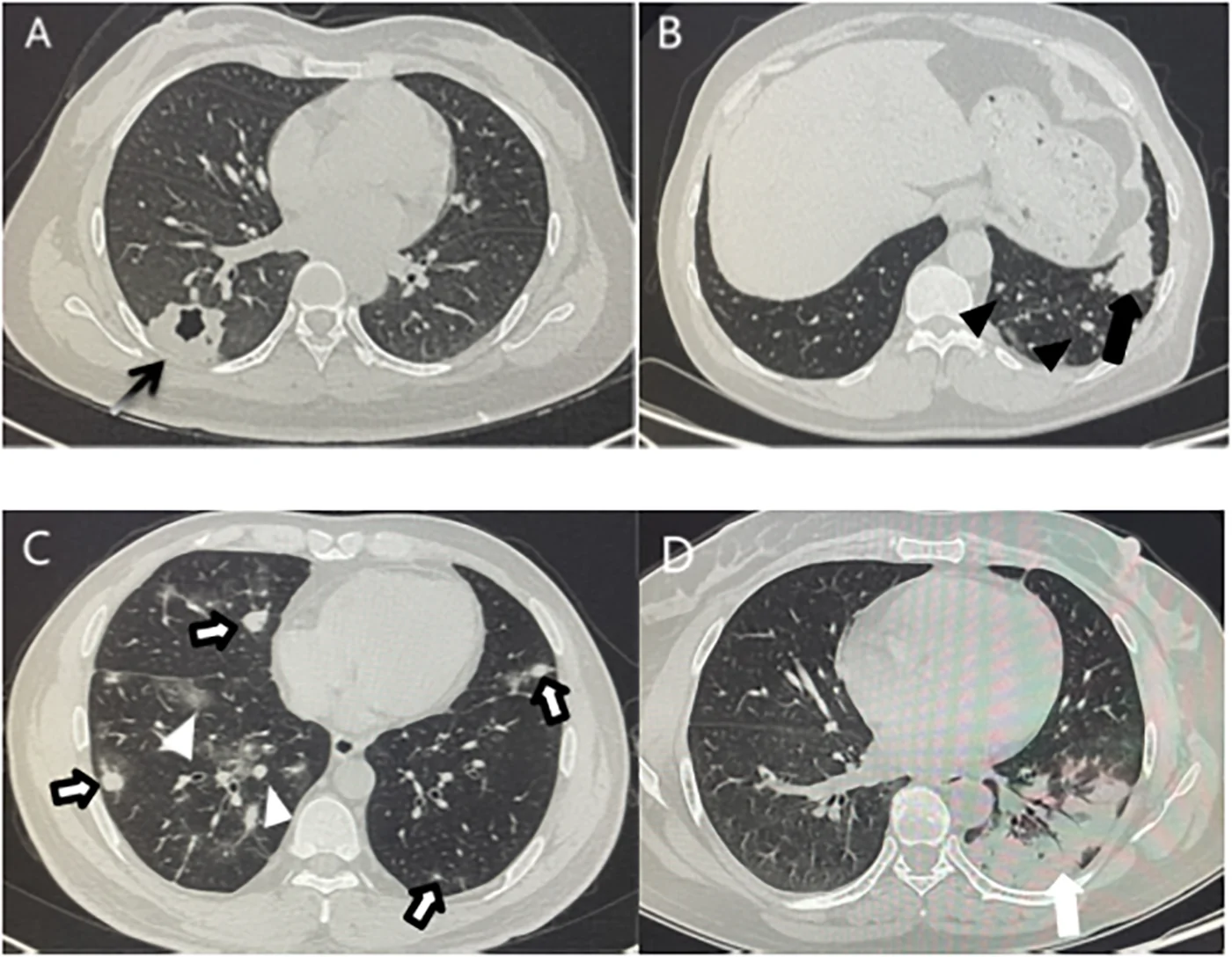
Representative chest CT images of pulmonary cryptococcosis. **(A)** Solitary nodule/mass type: A lobulated mass with internal cavitation (black arrow) in the right lower lobe. **(B)** Multiple nodules/masses type: Multiple irregular nodules (black arrowheads) and masses (black arrow) in the left lower lobe. **(C)** Mixed type: Multiple patches (white arrowheads) and nodules (black hollow arrows) distributed in both lungs, with some nodules surrounded by halo signs. **(D)** Patchy consolidation type: A large consolidation with air bronchogram (white arrow) in the left lower lobe.

Imaging manifestations were analyzed in 117 patients. The predominant pattern was nodular/mass type (93/117, 79.5%), comprising solitary nodules/masses (74/117, 63.2%) and multiple nodules/masses (19/117, 16.2%). Patchy consolidation (type III) was observed in 13 patients (11.1%), and mixed patterns (type IV) in 11 patients (9.4%) (Figure 2).

Lesions were predominantly unilateral and unilobar (106/117, 90.6%), with 63.3% located in the lower lobes. Peripheral distribution was observed in 86.3% of cases. Most lesions were <3.0 cm in size (94/117, 80.3%). Common morphological features included blurred margins (74/117, 63.3%), lobulation (80/117, 68.4%), spiculation (68/117, 58.1%), vascular convergence (66/117, 56.4%), and surrounding fibrotic strands (89/117, 76.1%). Characteristic signs included halo sign (29/117, 24.8%), cavitation (22/117, 18.8%), air bronchogram (19/117, 16.2%), satellite lesions (11/117, 9.4%), and punctate calcification (2/117, 1.7%). Among 37 patients with contrast-enhanced CT, mild and marked enhancement were observed in 20 (54.1%) and 6 (16.2%) cases, respectively. Pleural involvement occurred in 84 cases (71.8%), manifesting as broad-based pleural attachment (24/117, 20.5%) or pleural indentation (60/117, 51.3%).

As detailed in Table 3, following FDR correction, significant differences between ICP and NICP groups were maintained for four imaging features: solitary nodule/mass type (type I, Q = 0.030), patchy consolidation type (type III, Q = 0.035), solitary lesion (Q = 0.030), and numerous lesions (≥10 lesions, Q = 0.035). The halo sign, which showed a nominally significant *P*-value (0.035), did not remain significant after multiple comparison adjustment (Q = 0.105).

**Table 3:** Comparison of chest CT features of PC between ICP and NICP.

CT features	Total(*n* = 117)	ICP (*n* = 49)	NICP (*n* = 68)	χ²	*P*-Value	Q-Value
Type of lesions						
Single nodule/mass (type I)	74 (63.3%)	23 (46.9%)	51 (75.0%)	9.647	**0**.**002**	**0**.**030**
Multiple nodules/masses (type II)	19 (16.2%)	10 (20.4%)	9 (13.2%)	1.007	0.229	0.528
Patchy consolidation (type III)	13 (11.1%)	10 (20.4%)	3 (4.4%)	7.378	**0**.**007**	**0**.**035**
Mixed (type IV)	11 (9.4%)	6 (12.2%)	5 (7.4%)	0.800	0.371	0.598
Distribution of lesions						
Single lung	106 (90.6%)	44 (89.8%)	62 (91.2%)	0.318	0.573	0.678
Bilateral lungs	11 (9.4%)	5 (10.2%)	6 (8.8%)	0.064	0.801	0.829
Single lung lobe	101 (86.3%)	41 (83.7%)	60 (88.2%)	0.502	0.479	0.598
Multiple lung lobes	16 (13.8%)	8 (16.3%)	8 (11.8%)			
Location of lesions						
Right upper lobe	22 (18.8%)	7 (14.3%)	15 (20.1%)	1.127	0.288	0.587
Right middle lobe	15 (12.8%)	6 (12.5%)	9 (13.2%)	0.035	0.851	0.851
Right lower lobe	49 (41.9%)	25 (51.0%)	24 (35.3%)	2.894	0.089	0.296
Lef upper lobe	19 (16.2%)	5 (10.2%)	14 (20.6%)	2.258	0.133	0.399
Lef lower lobe	34 (29.1%)	16 (32.7%)	18 (26.5%)	0.528	0.467	0.599
Subpleural region	101 (86.3%)	41 (83.7%)	60 (88.2%)	0.502	0.479	0.599
No Subpleural region	16 (13.8%)	8 (16.3%)	8 (11.8%)			
Lesion size						
1–<3.0 cm	94 (80.3%)	36 (73.5%)	58 (85.3%)	3.297	0.069	0.259
≥3.0 cm	23 (19.7%)	13 (26.5%)	10 (14.7%)			
Number of lesions						
1	74 (63.2%)	23 (46.9%)	51 (75.0%)	9.647	**0**.**002**	**0**.**030**
2–10	24 (20.5%)	13 (26.5%)	11 (16.2%)	1.873	0.171	0.466
10∼	19 (16.2%)	13 (26.5%)	6 (8.8%)	6.565	**0**.**010**	**0**.**035**
Border of lesions						
Clear	43 (36.8%)	19 (38.8%)	24 (35.3%)	0.148	0.700	0.75
Blurred	74 (63.3%)	30 (61.2%)	44 (64.7%)			
Edges of lesion						
Smooth	34 (29.1%)	16 (32.6%)	18 (26.5%)	0.528	0.467	0.599
Roughness	83 (70.9%)	33 (67.4%)	50 (73.5%)			
Lobulation	80 (68.4%)	31 (63.3%)	49 (72.1%)	1.018	0.313	0.587
Spiculation	68 (58.1%)	28 (57.1%)	40 (58.8%)	0.543	0.461	0.599
Fibro stripe	89 (76.1%)	39 (81.3%)	50 (73.5%)	0.575	0.448	0.599
Vascular cluster sign	66 (56.4%)	29 (59.2%)	37 (54.4%)	0.264	0.608	0.702
Pleural relationship						
Wide substrate adhesion	28 (23.9%)	16 (32.7%)	12 (17.7%)	3.781	0.052	0.259
Pleural indentation sign	64 (54.7%)	28 (57.1%)	36 (52.9%)	0.203	0.652	0.724
Internal structure						
Cavity	22 (18.8%)	13 (26.5%)	9 (13.2%)	3.297	0.069	0.258
Air bronchogram	19 (16.2%)	10 (20.4%)	9 (13.2%)	1.077	0.299	0.587
Calcification	2 (1.7%)	0 (0%)	2 (2.9%)	1.466	0.226	0.528
Halo sign	29 (24.8%)	17 (34.7%)	12 (17.7%)	4.439	**0**.**035**	**0**.**210**
Satellite lesion	11 (9.4%)	6 (12.2%)	5 (7.4%)	0.800	0.371	0.599

ICP, immunocompromised patients; NICP, non-immunocompromised patients. Q-values were adjusted for multiple comparisons using the Benjamini–Hochberg false discovery rate (FDR) method. A Q-value < 0.05 was considered statistically significant and is shown in bold.

### 3.4 Relationship between serum indicators and CT imaging features in pulmonary cryptococcosis

Out of 31 patients who underwent serum CrAg testing, 26 cases (83.9%) showed positive results. Additionally, 45 patients underwent antigen titer testing for *Cryptococcus neoformans*, with 34 cases (75.6%) showing positive results. The distribution of imaging types with positive CrAg detection is as follows: type I 19.2% (5/26), type II 15.4% (4/26), type III 26.9% (7/26), and type IV 38.5% (10/26). The distribution of positive titers of *Cryptococcus neoformans* antigen in imaging is as follows: type I 29.4% (10/34), type II 23.5% (8/34), type III 17.6% (6/34), and type IV 29.4% (10/34). There was no statistically significant difference in the positive results of serum cryptococcal detection between the ICP and NICP groups (Table 4).

**Table 4:** Comparison of serum cryptococcal antigen detection between ICP and NICP groups.

Parameters	ICP(*n* = 49)	NICP(*n* = 68)	χ²	*P*-value
CrAg positive, *n* (%)	16 (80.0%, 16/20)	10 (90.9%, 10/11)	0.624	0.429
Follow-up CrAg titer positive, *n* (%)	21 (77.8%, 21/27)	13 (72.2%, 13/18)	0.180	0.671

ICP, immunocompromised; NICP, non-immunocompromised; CrAg, cryptococcal antigen. Follow-up CrAg titer refers to the serum cryptococcal antigen titer measured at the last available follow-up visit after treatment or surgical intervention.

**Figure 3:**
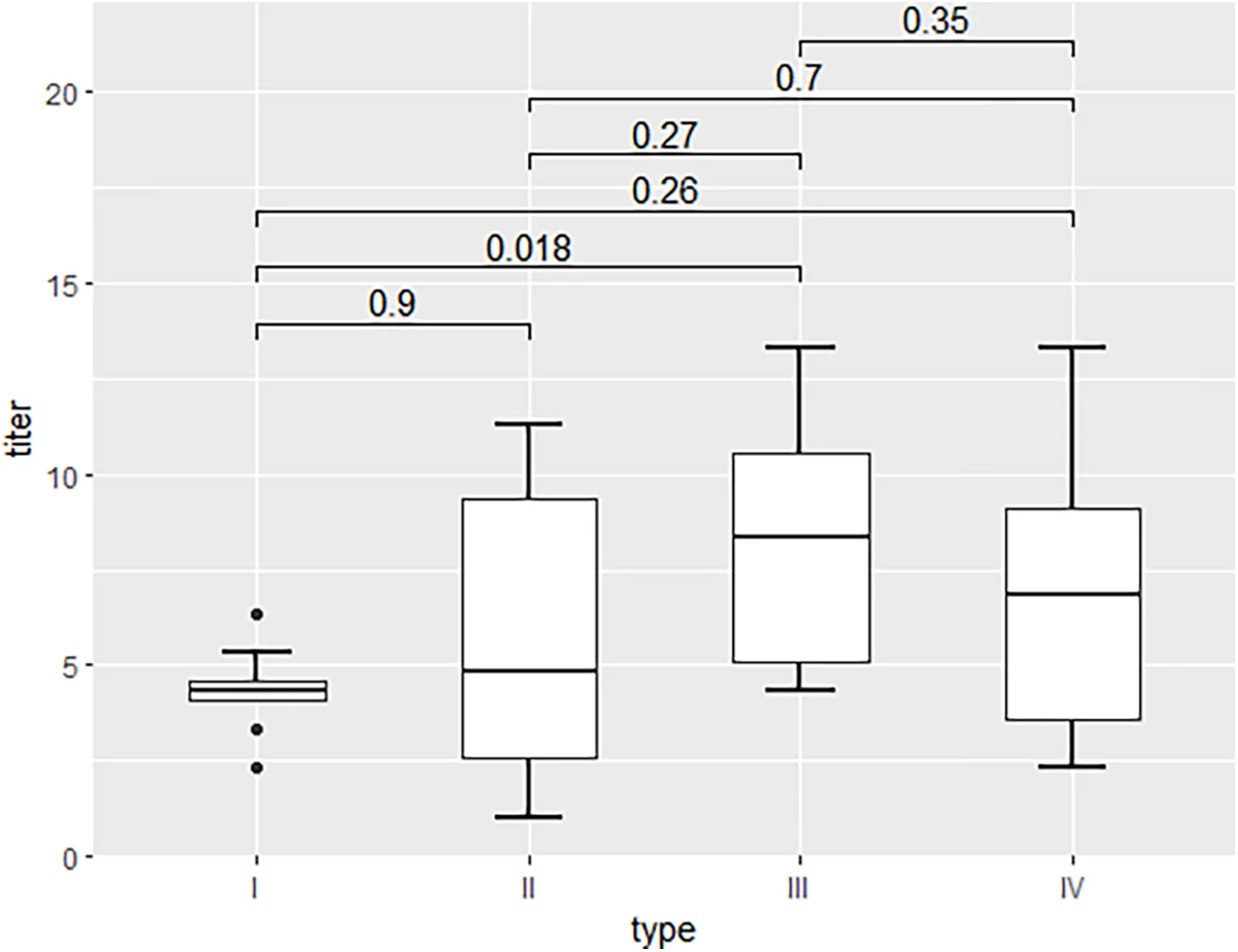
Relationship between serum cryptococcal antigen titer and CT imaging types.

To compare antigen titers across imaging types, titers were log2-transformed [log2(1/titer)] to approximate a normal distribution. The analysis revealed a statistically significant difference between type I and type III (*P* = 0.018), indicating that type III lesions were associated with significantly higher antigen titers than type I lesions. No significant differences were observed among the other imaging type comparisons (Figure 3).

The *y*-axis represents the log2-transformed reciprocal of the cryptococcal antigen titer [log2(1/titer)]. The *x*-axis represents the four CT imaging types (I–IV). A statistically significant difference was observed between type I and type III (*P* = 0.018); no significant differences were observed among the other type comparisons.

### 3.5 Treatment and outcomes

As of September 30, 2023, 113 out of 117 patients received treatment and underwent follow-up CT scans at our hospital. The specific treatment outcomes were as follows: ① Surgical treatment (65/113, 57.5%): Among these, 51 patients (44.7%) underwent surgery alone, consisting of immunocompetent, asymptomatic patients with solitary lesions smaller than 3 cm in diameter. None of these patients experienced disease recurrence or progression during follow-up. The remaining 14 patients (12.4%) received surgery combined with postoperative antifungal therapy, with the decision for adjuvant therapy based on the presence of positive surgical margins, multifocal disease, or immunocompromised status.; ② Antifungal therapy alone (48/113, 42.5%): This group included 42 patients (36.9%) receiving monotherapy and 6 patients (5.3%) receiving combination therapy. Monotherapy consisted of fluconazole (Diflucan) at 400 mg/day or voriconazole at 200 mg twice daily. Combination therapy consisted of an azole (fluconazole or voriconazole) combined with 5-flucytosine (1 g/day), reserved for patients with more severe disease, those who failed to respond to initial monotherapy, or those with suspected or confirmed central nervous system involvement. For comparative analysis, patients were categorized into two groups: “antifungal treatment” (including medical therapy alone and surgery combined with antifungal therapy) and “surgery alone without antifungal treatment.” This binary variable was incorporated as a potential confounding factor in our regression analyses. The medication duration ranged from 0.5 to 24 months, with an average of 7.7 ± 4.3 months. Among 48 patients treated with antifungal drugs alone, 24 patients were followed up for serum cryptococcal antigen titers. There was no statistically significant difference between the groups with or without immunocompromised conditions (*P* = 0.728).

**Figure 4:**
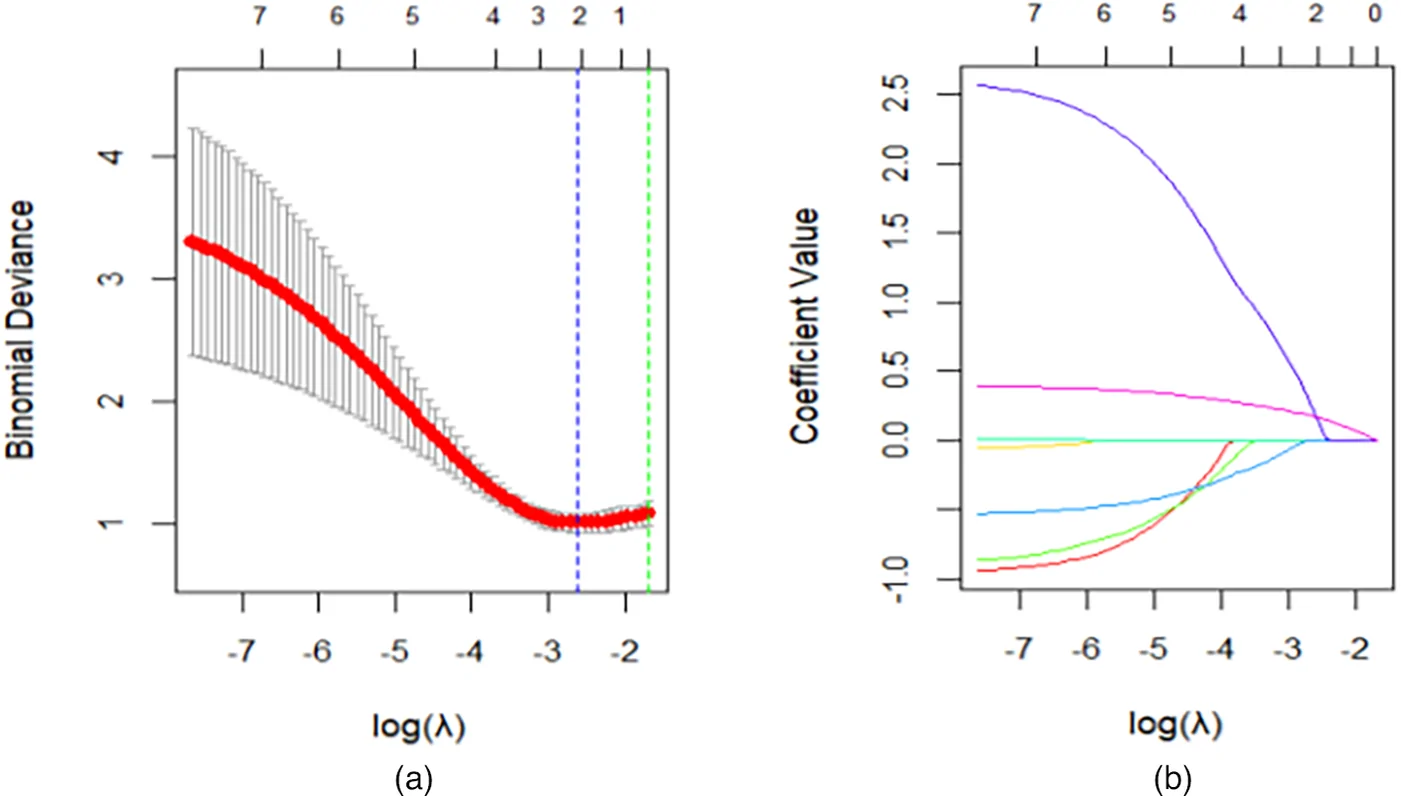
LASSO score of image absorption in pulmonary cryptococcosis after antifungal treatment. **(a)** Cross validation error plot showing the relationship between regularization parameter (log*λ*) and binomial bias. The red curve represents the average cross validation error, and the error bar represents one standard deviation. The green dashed line represents the optimal *λ* value selected by a standard error rule (*λ*. lse = 0.186, log*λ* = −1.68), while the blue dashed line represents the*λ*value with the minimum cross validation error (*λ*. min = 0.056, log*λ* = −2.89). Each curve in **(b)** represents a variable, including 1 (gender), 2 (age), 3 (clinical symptoms), 4 (cryptococcal antigen titer), 5 (presence of immunocompromised status), 6 (imaging type), and 7 (duration of antifungal drug use). Application of the one-standard-error rule (*λ*.1se) produced a null model with no predictors retained. Using the minimum error criterion (*λ*.min), three variables were selected as potentially associated with treatment response: presence of immunocompromised status, imaging type, and duration of antifungal drug use.

Follow-up CT scans were obtained for 31 patients after antifungal therapy. The degree of absorption (DOA) ranged from 30% to 99%. The mean DOA was 75.1% in immunocompromised patients (ICP, *n* = 21) and 86.9% in non-immunocompromised patients (NICP, *n* = 10). Distribution by imaging type revealed 11 type I, 9 type II, 9 type III, and 2 type IV cases, with corresponding mean DOA values of 76.2%, 72.7%, 87.2%, and 75.0%, respectively. LASSO regression was employed to identify factors associated with treatment response. Under the conservative lambda.1se (*λ*.1se) criterion, designed to mitigate overfitting, no variables showed robust associations with DOA. However, an exploratory analysis using the lambda.min (*λ*.min) criterion identified imaging type, presence of immunocompromised status, and duration of antifungal therapy as potential influencing factors (Figure 4). These latter findings are considered hypothesis-generating and require validation in larger cohorts.

## 4 Discussion

Cryptococcosis is a globally distributed invasive fungal infection that was first discovered in 1894. With the outbreak of the AIDS pandemic in the 1980s, cryptococcosis was considered a significant threat to human health (12). *Cryptococcus neoformans* is commonly found in soil, pigeon manure, and moldy vegetables and fruits, and is classified as an environmental saprophytic fungus. Cryptococcus neoformans *Cryptococcus neoformans* isolated from pigeon droppings is considered the primary source of pulmonary cryptococcal infection (13). As reported by Xie et al. (14), 12.19% of patients had a history of direct exposure to pigeon droppings. In this study, only 2 cases (1.7%) had a history of related contact, suggesting that the possibility of PC cannot be ruled out even in the absence of environmental exposure factors. *Cryptococcus neoformans* and *Cryptococcus gattii* are the most common causes of human infection (15). It was previously believed that PC was more common in patients with immunocompromised conditions. In recent years, the incidence rate of PC in the immunocompetent population has been rising. In this study, 58.12% of patients had no known immune deficiency, which is consistent with previous reports (4, 12, 16). This suggests that pulmonary shadows should not be excluded from PC even though patients without immune deficiency.

Among the 117 patients with pulmonary cryptococcosis (PC), 59.0% (69/117) were asymptomatic at presentation, a finding consistent with the known high frequency of subclinical infection in this disease (17). The remaining 41.0% (48/117) exhibited non-specific respiratory symptoms including cough, sputum, and dyspnea. Initial univariate analysis showed differences in the incidence of symptoms such as cough, asthma and dyspnea between the two groups (*P* < 0.05). However, after False Discovery Rate correction for multiple comparisons, these differences were no longer statistically significant (all Q > 0.05). This indicates that while a trend toward increased symptom burden may exist in ICP patients (18), the baseline clinical presentation does not reliably differentiate between immune status groups. The similar symptomatic profile may reflect comparable pulmonary tissue response to cryptococcal infection regardless of host immunity, though different imaging patterns may be associated with varying clinical manifestations (19). These findings confirm that clinical symptoms alone have limited value in predicting underlying immune status in PC patients (17), supporting the need for laboratory and radiographic evaluation for accurate stratification, as emphasized in recent diagnostic guidelines (20) and supported by advances in diagnostic techniques like metagenomic sequencing (21). The laboratory profile provides objective differentiation, with immunocompromised (ICP) patients showing significantly lower red blood cell counts and albumin levels after FDR correction (all Q < 0.05), a finding consistent with the known effects of chronic disease burden on nutritional and hematological homeostasis (22). The absence of significant differences in routine inflammatory markers (23), coupled with uniformly negative results for other opportunistic infections (24), suggests that the pulmonary inflammatory response to *Cryptococcus* may be compartmentalized and relatively independent of the systemic immune status. The low detection yield of conventional microbiological methods (e.g., sputum culture, CSF antigen) in our cohort aligns with their recognized limited sensitivity for diagnosing pulmonary cryptococcosis (24), underscoring the necessity of incorporating advanced diagnostic approaches such as antigen testing and molecular methods as recommended by current guidelines (22).

The imaging spectrum of pulmonary cryptococcosis (PC) in our cohort, encompassing solitary/multiple nodules, masses, and consolidation, aligns with established descriptions. Our analysis confirms that the nodular/mass type (Type I) is the most prevalent presentation (79.5%), with the solitary form (Type I) being significantly more common in non-immunocompromised (NICP) patients (Q = 0.030). Conversely, complex patterns including patchy consolidation (Type III) and numerous lesions (≥10) demonstrated a significant association with immunocompromised (ICP) status after rigorous multiple comparison adjustment (Q = 0.035 for both), corroborating findings in previous literature (25, 26) that more advanced or disseminated imaging patterns are frequent in ICP hosts. The predominant peripheral (86.3%) and lower lobe (63.3%) distribution of lesions supports the hypothesis that the small size of cryptococcal spores facilitates deposition and implantation in the distal airways and alveoli after inhalation (27). Most lesions were small (<3.0 cm, 80.3%), with common malignant-mimicking features such as lobulation (68.4%), spiculation (58.1%), and vascular convergence (56.4%) observed at the periphery. The high frequency of surrounding fibrotic strands (76.1%) suggests a host tissue reaction attempting to contain the infection. Characteristic signs such as the halo sign (24.8%), while valuable as an indicator for differentiating fungal infection from malignancy or tuberculoma and consistent with prior reports (25, 28), did not remain a statistically significant differentiator between ICP and NICP groups after FDR correction (*P* = 0.035, Q = 0.105). This indicates that while the halo sign is a common feature of PC, its presence is not robustly tied to the host's immune status in this cohort. Other internal characteristics, including cavitation (18.8%) and air bronchogram (16.2%), were observed without significant intergroup differences.

These imaging characteristics—particularly the predominance of small, well-circumscribed solitary nodules in immunocompetent patients—provide the radiological basis for considering surgical management in carefully selected cases. Indeed, a noteworthy finding in our cohort is the subgroup of 51 patients (44.7%) who underwent surgery alone. All were immunocompetent, asymptomatic individuals with solitary lesions <3 cm, and none experienced recurrence or progression during follow-up. This observation is consistent with previous studies demonstrating that surgical resection alone achieves favorable outcomes with no recurrence in immunocompetent patients with limited pulmonary cryptococcosis (29, 30). In carefully selected patients, surgery alone is curative and can avoid the side effects, costs, and drug interactions of prolonged antifungal therapy, and should not be perceived as a treatment deficiency. However, the absence of events precluded a meaningful Kaplan–Meier analysis, and prospective studies with standardized follow-up are needed to confirm the durability of this approach. Serum cryptococcal antigen (CrAg) is a cornerstone in diagnosing cryptococcosis, exhibiting high accuracy when combined with clinical and imaging findings. In our cohort, 83.9% (26/31) of tested patients were CrAg-positive, consistent with prior reports (31). The antigen titer correlates with disease burden, with the highest pre-treatment titer reaching 1:10240. Following antifungal therapy, a significant titer reduction was observed in all 24 followed-up patients, with 37.5% (9/24) seroconverting to negative. However, persistent antigen positivity does not invariably indicate treatment failure. Consistent with guidelines (32) and a large retrospective study (31), 54.2% (13/24) of our patients showed significant imaging improvement despite non-negative titers at discontinuation. Therefore, clinical efficacy should be assessed comprehensively, integrating symptom resolution, radiographic changes, and laboratory trends, rather than relying on CrAg titer normalization alone.

After antifungal treatment, 31 cases in this study underwent follow-up chest CT scans. The lesions showed a decrease in size, with a Degree of Absorption (DOA) ranging from 30% to 99%. Univariate analysis indicated that the average DOA of the immunocompromised patients (ICP, 75.1%) was significantly lower than that of the non-immunocompromised patients (NICP, 86.7%) (*P* = 0.038). This difference may be attributed to the stronger ability of patients with normal immune function to eliminate *Cryptococcus* after treatment. Among the different imaging types, DOA was also significantly higher in type III (patchy consolidation) than in other types. This type is primarily characterized by mucinous degeneration and accumulations of cryptococcal bodies but lacks granulomatous reactions, which may facilitate its easier absorption after medication (33). However, when these factors were evaluated together in a multivariate LASSO regression model—applied with conservative criteria to address overfitting in our limited sample—no robust predictors could be identified. Notably, an exploratory analysis using a less stringent criterion nominated lesion imaging type, immunocompromised status, and treatment duration as the most promising candidate factors emerging from our dataset. This discrepancy underscores the instability of model selection in small samples; while these exploratory findings lack definitive statistical support, they provide valuable, hypothesis-generating insights that merit prioritization in future large-scale validation studies.

Several important limitations of this study must be acknowledged. First, the single-center retrospective design may limit the generalizability of our findings and introduce selection bias. Second, although we optimized the criteria for defining immunocompromised status, we lacked precise stratification by severity of immunosuppression, which may obscure clinically meaningful heterogeneity within the immunocompromised group. Third, the small follow-up cohort (*n* = 31) substantially reduces statistical power and model stability, as reflected by the failure of the conservative LASSO regression to identify robust predictors of lesion absorption. Fourth, the heterogeneity of antifungal treatment regimens—including variations in drug selection, dosage, and duration—represents a potential confounding factor. Although we incorporated treatment status as a binary variable in our regression models to adjust for this effect, the lack of standardized protocols limits our ability to precisely evaluate the influence of antifungal therapy on clinical outcomes. Fifth, routine species differentiation of Cryptococcus isolates was not implemented clinically throughout the study period, precluding investigation of the association between pathogen species and host immune status. Sixth, the retrospective nature of data collection precluded standardized serial CrAg titer measurements, limiting our ability to assess the relationship between duration of antigenemia and quantitative antigen titers. Finally, the absence of events (recurrence or progression) in the 51 patients treated with surgery alone precluded a meaningful Kaplan–Meier survival analysis, and the limited sample size of this subgroup restricts the generalizability of our findings. Future prospective studies with larger cohorts and standardized follow-up schedules are needed to validate our findings and address these limitations.

## Conclusion

In conclusion, this study delineates distinct clinical profiles of pulmonary cryptococcosis (PC) in immunocompromised (ICP) and non-immunocompromised (NICP) hosts. While the overall clinical presentation, including non-specific respiratory symptoms, proved unreliable for differentiating immune status, objective laboratory and radiographic markers provided critical insights. ICP patients exhibited a significant hematological and nutritional burden, characterized by lower red blood cell counts and albumin levels. Radiologically, ICP status was strongly associated with more complex and disseminated disease patterns, including patchy consolidation and numerous lesions, whereas solitary nodules were more characteristic of NICP patients.

The diagnosis of PC remains challenging due to the high rate of asymptomatic presentation and the limited sensitivity of conventional microbiological methods. Our findings strongly advocate for a comprehensive diagnostic strategy that integrates serum cryptococcal antigen testing with detailed chest CT evaluation. Furthermore, the assessment of treatment response should not rely solely on the normalization of antigen titers but must incorporate serial imaging and clinical improvement, as antigen persistence is common despite radiographic resolution. The instability of our multivariate prognostic model underscores the profound impact of limited sample size and highlights the imperative for future large-scale, prospective studies to identify robust predictors of treatment outcome and validate the exploratory associations identified herein.

From a clinical perspective, two key messages emerge from our findings. First, given that the majority of cases were incidentally discovered in asymptomatic individuals, population-based screening is not recommended; instead, diagnostic vigilance during routine imaging should be emphasized. Second, in immunocompetent patients with small solitary lesions, surgery alone is an effective option that can reduce the need for prolonged antifungal therapy.

## Data Availability

The raw data supporting the conclusions of this article will be made available by the authors, without undue reservation.
